# A response to “Realism and robustness require increased sample size when studying both sexes”

**DOI:** 10.1371/journal.pbio.3002578

**Published:** 2024-04-12

**Authors:** Benjamin Phillips, Timo N. Haschler, Natasha A. Karp

**Affiliations:** 1 Data Sciences & Quantitative Biology, Discovery Sciences, R&D, AstraZeneca, Cambridge, United Kingdom; 2 Bioscience Renal, Research and Early Development Cardiovascular, Renal and Metabolism, Biopharmaceutical R&D, AstraZeneca, Cambridge, United Kingdom

In our original paper [1], we concluded that inclusion of both sexes does not require an increase in sample size by default. Drobniak and colleagues [2] have helpfully highlighted an exception to this general rule, in cases where the variability for a trait differs between the sexes. In these situations, the statistical analysis needs amending to account for the unequal variance and there is a potential power penalty [2]. We would argue however that the general rule still applies, unless there is empirical evidence on a case-by-case basis that suggests that a more complex bespoke power calculation is required, which can then guide the subsequent required sample size.

Drobniak and colleagues [2] assert that “heteroscedasticity between two sexes should be the norm rather than the exception” and provide 2 arguments for this position. Firstly, they cite 2 meta-analyses. We note that the conclusions, within these papers, do not support the default assumption of heteroscedasticity. Indeed, the opposite position (assumption of homoscedasticity) is better supported. For example: “For the average trait of interest to animal researchers, variability does not differ between males and unstaged females” [3] and “the distributions (*diff in CV*) [explanation added] were extremely leptokurtic, so that many differences were close to 0” [4]. Furthermore, additional studies, be it clinical data [5], behaviour metrics [6], microarray data [7], or data from rats [8] have found similar results. In summary, the literature supports the position that unless there is evidence to the contrary, assume equal variance.

Secondly, Drobniak and colleagues [2] argue that the variance between the sexes will inevitably differ due to Taylor’s law, where the mean and the standard deviation correlate. Consequently, baseline sex differences will inherently lead to variance differences. It is important to note that, for traits that follow Taylor’s law, then any intervention effect (e.g., drug treatment) will also lead to a change in variance between treatment groups. In these situations, it would be inappropriate to fit a standard factorial analysis and the associated statistical power would be irrelevant. As we stated in our original paper [1], it is important to ensure the assumptions hold for your chosen statistical analysis. In biological situations where the mean and variance correlate, a variance stabilising transformation (e.g., log) is an easy applicable strategy to try which should improve the characteristics and allows the application of a standard statistical strategy.

In conclusion, we believe that our original advice still holds. If you have no specific knowledge to the contrary, it is reasonable to proceed assuming homoscedasticity, with no need to increase the overall sample size. If evidence of sex-related heteroscedasticity in the measure of interest is apparent or later emerges, it is important to adapt the statistical analysis plan and power calculation accordingly. As inclusion of both sexes increases, we will start to have the data to conduct more nuanced planning.
